# Association Between Helicobacter pylori and Steatosis Severity on Transient Elastography

**DOI:** 10.7759/cureus.34042

**Published:** 2023-01-21

**Authors:** Andre Fialho, Andrea Fialho, Bruno Ribeiro, Miguel Malespin, Silvio De Melo, Ron Schey, Peter Ghali

**Affiliations:** 1 Internal Medicine: Gastroenterology, South Central Regional Medical Center, Laurel, USA; 2 Gastroenterology, University of Florida College of Medicine – Jacksonville, Jacksonville, USA; 3 Gastroenterology and Hepatology, Tampa General Hospital, Tampa, USA; 4 Gastroenterology, Orlando Veterans Affairs Medical Center, Orlando, USA

**Keywords:** fatty liver, hepatic fibrosis, hepatic steatosis, transient elastography, helicobacter pylori

## Abstract

Background

A possible association between *Helicobacter pylori* (HP) infection and liver diseases including steatosis is suspected. There is a lack of studies evaluating the association of HP and liver steatosis severity using transient elastography.

Aim

The aim of this study was to evaluate the frequency and risk factors for liver steatosis measured by transient elastography in patients with or without HP.

Methods

A total of 484 patients tested for liver steatosis and fibrosis using transient elastography from January 2017 to June 2018 were evaluated. Ninety-one patients who were also tested for *H. pylori* infection were included in the study. Transient elastography findings were compared between HP-positive patients and HP-negative patients. Demographic, clinical, and laboratory variables and the presence and severity of liver fibrosis and steatosis were analyzed.

Results

Patients with HP had a higher frequency of steatosis on transient elastography (86.8% *vs.* 60.7%, p =0.009). Patients with HP had increased steatosis severity compared to HP-negative patients (mild steatosis 15.8% *vs.* 7.1%, p=0.037; moderate to severe steatosis 71.1% *vs.* 53.6%, p=0.015, respectively). In the stepwise multivariate logistic regression analysis, HP infection remained an independent risk factor for steatosis (odds ratio: 4.36, 95% confidence interval: 1.09-14.78; p=0.037).

Conclusion

Patients with HP had an increased steatosis frequency, and patients with liver steatosis may warrant HP evaluation and treatment.

## Introduction

Approximately half of the world population is infected by *Helicobacter pylori* (HP), a Gram-negative bacterium which causes chronic gastritis and peptic ulcer disease [1]. In addition, HP infection has been associated with a variety of extra-gastric conditions, and great interest exists in studies showing an association of this bacterial pathogen with liver disorders [2,3].

Liver steatosis is characterized by excessive deposition of fat in the liver cells and is usually a consequence of metabolic syndrome, alcohol and/or medication use. Non-alcoholic fatty liver disease (NAFLD) is defined by the presence of hepatic steatosis regardless of inflammatory changes and/or fibrosis. Non-alcoholic steatohepatitis happens when liver steatosis is superimposed with significant inflammation and cell apoptosis. Liver steatosis is one of the leading causes of chronic liver disease in Western counties [4,5]. Transient elastography is a non-invasive method with excellent accuracy in quantifying the levels of hepatic steatosis in patients at risk for liver disease [6].

The association between HP infection and the severity of liver steatosis on liver elastography is poorly understood. We hypothesized that the severity of liver steatosis may be associated with the presence of HP infection. Therefore, the aim of this study is to investigate the risk factors associated with liver steatosis in patients infected with HP.

## Materials and methods

In this study, we retrospectively assessed the electronic medical record of 484 consecutive patients from the Gastroenterology Lab Data Bank at the University of Florida Jacksonville who had been submitted to transient liver elastography for the diagnosis of liver conditions from January 2017 to June 2019. Extensive demographic and clinical variables were collected, and patients tested for HP infection were included in this study. The Institutional Review Board of the University of Florida Jacksonville Institutional approved this study (IRB number 201601518).

Inclusion and exclusion criteria

Inclusion criteria were adult patients aged ≥ 18 years old with 1) transient elastography and 2) esophagogastroduodenoscopy gastric biopsies or stool antigen testing for the diagnosis of HP. Exclusion criteria were prior treatment for HP, no HP evaluation, and the presence of interquartile range (IQR) >30% on liver elastography.

Study and control groups

Ninety-one patients of the initial 484 patients submitted to transient elastography fulfilled the inclusion criteria and were enrolled in the study. The 91 patients were subdivided into two groups: a study group composed of HP-positive (n=37) patients and a control group composed of HP-negative (n=54) patients. 

Study variables

A total of 18 variables were studied. Clinical variables included age, gender, body mass index (BMI), obesity (defined as BMI ≥30), hypertension (HTN), hyperlipidemia (HLD), diabetes mellitus (DM), metabolic syndrome, and signs of portal hypertension (history of ascites, esophageal varices and/or hepatic encephalopathy). The laboratory variables included in this study were aspartate aminotransferase (AST), alanine aminotransferase (ALT), total bilirubin, alkaline phosphatase, and albumin. The transient elastography measurements included in the study were liver stiffness measured in kPa and liver steatosis.

Diagnostic criteria

Transient elastography data collection was performed on individuals lying in a supine position which appropriately spread the right rib cage by elevating the right arm and crossing the right lower extremity over the left one [6]. After appropriate gel lubrification, the elastography probe was placed perpendicular to the 9th to 11th intercostal spaces close to the right lobe of the liver at the middle axillary line. Measurements of velocity and the intensity of attenuation of the so-called shear wave were collected, and data were processed automatically and electronically calculated through calibrated FibroScan® software (echosens, Milan, Italy). Fibrosis is expressed in kilopascals (kPa) and the steatosis in controlled attenuation parameter (CAP) score measured in decibels per meter (dB/m) [6].

Outcome measurements

The primary outcomes of this study were to assess the frequency and severity of liver steatosis in patients with HP and to evaluate the association between HP and liver steatosis on transient elastography. CAP values range from 100 to 400 dB/m with more severe steatosis being indicated by higher numbers. The cut-off CAP values for no/minimal steatosis (S0) was < 219.5 dB/m, mild steatosis (S1) was 219.5 to 230 dB/m, moderate steatosis (S2) 230 to 283 dB/m, and severe steatosis (S3) >283 dB/m [7].

Statistical analysis

All statistical analyses were performed using IBM SPSS Statistics for Windows, Version 22 (Released 2013; IBM Corp., Armonk, New York, United States). Mean ± standard deviation (SD) or N% was used to present continuous variables. Potential variables associated with HP infection were identified by univariable analysis. Continuous factors were analyzed using Student t-tests or the non-parametric Wilcoxon rank sum test categorical variables were analyzed using Pearson chi-square tests. Risk factors associated with liver steatosis in HP-positive patients were assessed by multivariate logistic regression analysis. A p<0.05 was considered statistically significant. 

## Results

The medical records of 484 patients submitted for transient liver elastography were reviewed. Of these, 91 patients who had *H. pylori* tested were included for further analysis. Patients were subdivided into *H. pylori* positive (n=37, the study group) or *H. pylori* negative (n=54, the control group).

No difference was found between HP-positive and HP-negative patients with regard to age (57.4±8.6 vs. 57.6±10.9; P=0.212), male sex (42.1% vs. 39.3%; P=0.475), HTN (63.2% vs. 60.7%; P=0.492), DM (39.5% vs. 37.5%; P=0.508), HLD (34.2% vs. 33.9%; P=0.575), BMI (29.5±1.2 vs. 28.1±0.8; P=0.561), metabolic syndrome (89.5% vs. 80.4%; P=0.186), AST (29.8±3.18 vs. 31.81±2.9; P=0.497), ALT (34.1±3.9 vs. 31.8±3.1;P=0.542), total bilirubin (0.51±0.03 vs. 0.53±0.06; P=0.172), alkaline phosphatase (88.4±6.1vs.85.2±4.8; P=0.844), albumin (4.2±0.05 vs 4.2±0.04; P=0.898), signs of portal hypertension with history of ascites, esophageal varices MO and/or hepatic encephalopathy (7.9% vs. 1.8%; P=0.179), IQR score (17.1±1.2 vs. 15.9±1.1; P=0.343), and liver stiffness EkPA (9.0±1.6 vs. 7.4±0.8; P=0.431) (Table 1).

**Table 1:** Bivariate analysis of patients with and without H. pylori HTN, hypertension; DM, diabetes mellitus; HLD, hyperlipidemia; BMI, body mass index; Metabolic Sd, metabolic syndrome; AST, aspartate aminotransferase; ALT, alanine transaminase; CAP, controlled attenuation parameter; dB/m, decibels per meter; kPa, kilopascals; IQR, interquartile range score; S, steatosis; F, fibrosis

Variables	H.pylori + (N=37)	H. pylori – (N=54)	p-value
Age	57.4±8.6	57.6±10.9	0.212
Male	16 (42.1%)	22 (39.3%)	0.475
HTN	24 (63.2%)	34 (60.7%)	0.492
DM	15 (39.5%)	21 (37.5%)	0.508
HLD	13 (34.2%)	19 (33.9%)	0.575
BMI	29.5±1.2	28.1±0.8	0.561
Metabolic Sd	34 (89.5%)	45 (80.4%)	0.186
AST	29.8±3.18	31.81±2.9	0.497
ALT	34.1±3.9	31.8±3.1	0.542
Total bilirubin	0.51±0.03	0.53±0.06	0.172
Alkaline phosphatase	88.4±6.1	85.2±4.8	0.844
Albumin	4.2±0.05	4.2±0.04	0.898
Portal hypertension	3 (7.9%)	1 (1.8%)	0.179
CAP score (dB/m)	281.9±10.4	262.2±9.2	0.398
Liver stiffness measurement (kPa)	9.0±1.6	7.4±0.8	0.431
S score			
S0	5 (13.2%)	22 (39.3%)	0.034
S1	6 (15.8%)	4 (7.1%)	
S2	8 (21.1%)	9 (16.1%)	
S3	19 (50.0%)	21 (37.5%)	
F score			
F0	22 (57.9%)	38 (67.9%)	0.612
F1	1 (2.6%)	2 (3.6%)	
F2	1 (2.6%)	5 (8.9%)	
F3	3 (7.9%)	4 (7.1%)	
F4	4 (10.5%)	7 (12.5%)	

In the univariate analysis, patients with HP had increased steatosis severity compared to HP-negative patients (mild steatosis 15.8% vs. 7.1%, p=0.037; moderate to severe 71.1% vs. 53.6%, p=0.015, respectively). Male sex, HP, HTN, DM, HLD, metabolic syndrome, and liver steatosis severity were included in the final model of the multivariate logistic regression analysis. Infection with HP remained an independent risk factor for steatosis after adjusting for all other variables in the model (odds ratio (OR): 4.36, 95% confidence interval (CI): 1.09-14.78; p=0.037) (Table 2).

 

**Table 2:** Multivariate analysis of potential risk factors for H. pylori HTN, hypertension; DM, diabetes mellitus; HLD, hyperlipidemia; Metabolic sd, metabolic syndrome; S, steatosis; CI, confidence interval; OR, odds ratio.

Variables	OR	95% CI	R square	p-value
Male	0.59	0.27-1.76	0.593	0.443
HTN	0.21	0.72-1.25	0.121	0.728
DM	1.01	0.34-2.88	0.010	0.997
HLD	1.16	0.39-3.42	0.077	0.781
Metabolic sd	0.65	0.11-3.63	0.244	0.623
S score	4.36	1.09-14.78	7.047	0.037

## Discussion

In the present study, patients with HP had increased steatosis severity compared to HP-negative patients regardless of metabolic syndrome. No association between increased liver stiffness and HP was found. HP infection is most commonly a life-long infection that is acquired in childhood. Given the persistence of HP infection for many years, it has potential metabolic consequences unless treated with antibiotics [8].

Studies have shown that HP infection induces the release of pro-inflammatory cytokines such as tumor necrosis factor alpha, interferon gamma, interleukin (IL)-1, IL-6, IL-8, IL-10, IL-12, and C reactive protein and was shown to be involved in the pathogenesis of insulin resistance [9,10]. To our knowledge, there is a scarcity of studies evaluating HP infections and liver elastography findings. Prior to the advent of liver elastography, invasive liver biopsy was required for liver fibrosis and steatosis quantification. Liver elastography has emerged in clinical practice as an important tool for the evaluation of liver steatosis and fibrosis.

In a similar study published in 2021, Liu et al. have not found HP to be significantly associated with NAFLD or liver steatosis, whereas in this study, a possible association of *H. pylori* with elevated liver stiffness in men was suspected instead [11]. Populational characteristic variations can possibly influence this difference in findings as the overall BMI in the Chinese population studied was found to be much lower than the one found in the southern US one studied in our manuscript. In addition, although no association between increased liver stiffness and HP was found in our paper, a possible role of HP infection in liver fibrosis development has been postulated priorly and is consistent with prior animal studies on liver cirrhosis that found immunohistochemical HP-positive antigen fragments in the liver of fibrotic liver specimens, suggestive that HP can have a role in liver fibrosis deposition [12]. The chronic HP infection generates a state of inflammation [13]. We postulate that this can synergistically predispose to a more intense fat deposition in the liver in patients with classical risk factors such as obesity and metabolic syndrome.

Our study has limitations. First, the power of the study may have been compromised by the small number of patients with liver steatosis. There is a non-statistical difference in BMI between HP positive and negative groups, which potentially could not be appreciated due to study sample size and power limitations; however, in both groups, BMI levels were >25 and below 30, consistent with overweight and no obvious clinical confounding was noted as if one of the groups were in different BMI classification ranges. Second, since the study was conducted in a tertiary center, it is possible that selection and referral bias occurred and patients in our study may not have been representative of the general population as they may have had more severe disease. In addition, because this was a retrospective study, it was not possible to evaluate other risk factors for HP or the inflammatory cytokines associated with liver steatosis. Future prospective studies with larger sample sizes are necessary to confirm a cause-and-effect association between HP infection and the degree of liver steatosis.

## Conclusions

In conclusion, patients with HP had increased steatosis frequency and severity when compared to HP-negative patients. This may be due to a pro-inflammatory state induced by the HP infection, which promotes the secretions of cytokines that possibly influence fat deposition in the liver. No association with increased liver stiffness was found in our study. Patients with risk factors for liver steatosis may warrant HP evaluation and treatment given potential implications on disease severity. Further prospective studies are necessary to confirm this association.
